# Prolonged social isolation promotes depressive-like behavior in male and female mice, with sex-related differences in the stress response

**DOI:** 10.1186/s13293-026-00874-0

**Published:** 2026-03-12

**Authors:** Yamila Cepeda, Catalina Tobar, Naoko Jara, Claudio Carril-Pardo, Andrés Villarroel, Raúl Lagos, Antonia Recabal, Estefanía Nova-Lamperti, Ana María Obregón-Rivas, Pía M. Vidal, Roberto Elizondo-Vega, Karina Oyarce

**Affiliations:** 1 Laboratorio de Neuroinmunología, Departamento de Bioquímica Clínica e Inmunología, Facultad de Farmacia, Universidad de Concepción, Concepción, Chile; 2 Departamento de Ciencias Biológicas y Químicas, Facultad de Ciencias, Universidad San Sebastián, sede Concepción, Campus Tres Pascualas, Concepción, Chile; 3 Laboratorio de Biología Celular, Departamento de Biología Celular, Facultad de Ciencias Biológicas, Universidad de Concepción, Concepción, Chile; 4 Faculty of Pharmacy, Department of Clinical Biochemistry and Immunology, Universidad de Concepción, Concepción, Chile; 5 Escuela de Nutrición y Dietética, Facultad de Ciencias de la Rehabilitación y Calidad de Vida, Universidad San Sebastián, Concepción, Chile; 6 Neuroimmunology and Regeneration of the Central Nervous System Unit, Biomedical Science Research Laboratory, Department of Basic Sciences, Faculty of Medicine, Universidad Católica de la Santísima Concepción, Concepción, Chile

**Keywords:** Social isolation, Chronic stress, Dimorphism, Neuroinflammation, Depressive-like behavior

## Abstract

**Background:**

Social isolation is a chronic psychological stressor with high translational relevance to depression in humans, particularly in the aftermath of the COVID-19 pandemic. However, few preclinical studies have evaluated its sex-dependent effects. Some studies have shown that after four weeks of social isolation, only males exhibit depressive-like behavior, without a comprehensive view of the underlying immune and neuroimmune alterations.

**Methods:**

Here, we examined the impact of prolonged social isolation on depressive- and anxiety-like behaviors of adult male and female mice, using the forced swim, splash, open field, and light/dark box tests. We also analyzed peripheral immune profiles through flow cytometry, ELISA and qRT-PCR, and neuroimmune responses through qRT-PCR and immunofluorescence for astrocytes and microglia.

**Results:**

After seven weeks of social isolation, both males and females exhibited depressive-like behavior and inflammatory signs such as elevated neutrophils in circulation, decreased IL-10 expression in the spleen, higher expression of IDO in the hippocampus, and higher microglia number. However, sex-related differences were also detected. Isolated males show lower body weight, with no changes in corticosterone levels, while isolated females exhibit increased corticosterone levels, higher IL-1β expression in the hippocampus, and higher microglia total area.

**Conclusions:**

After seven weeks of social isolation, both sexes exhibit depressive-like behavior, with sex-related differences in body weight, corticosterone levels, and cellular and molecular signs of neuroinflammation. These findings highlight the importance of temporality and sex as key variables in the behavioral and physiological responses to chronic stress. Given the increased prevalence of depression in women, these results provide new insights into sex-specific susceptibility to chronic stress and may inform the development of tailored diagnostic and therapeutic strategies.

**Supplementary Information:**

The online version contains supplementary material available at 10.1186/s13293-026-00874-0.

## Plain language summary

Social isolation is a chronic psychological stressor known to increase the risk of depression in humans, and it can be used to model depressive-like behavior in mice to better understand the physiopathology of this mental illness. Most of the available literature on chronic stress models, including social isolation, uses only males to characterize biological alterations and propose novel therapeutic strategies, without considering hormonal, immune and brain differences. The studies that incorporate females have not observed alterations at shorter times of social isolation, giving the impression that females are resistant to this type of stress. In this study, we show that at longer times of stress exposure, females do show depressive-like behavior and, unlike males, do not experience body weight loss, have higher levels of corticosterone, and more signs of neuroinflammation. These findings highlight the importance of temporality and sex as key variables in the behavioral and physiological responses to social isolation.

**Supplementary Information:**

The online version contains supplementary material available at 10.1186/s13293-026-00874-0.

## Highlights

- Prolonged social isolation (seven weeks) induces depressive-like behavior in both male and female mice.
- Although both sexes increase their food intake when socially isolated, only males lower their body weight.
- Both sexes show increased frequencies in circulating neutrophils.
- Only females show higher corticosterone levels in plasma and higher microglia complexity in response to chronic social isolation.

**Supplementary Information:**

The online version contains supplementary material available at 10.1186/s13293-026-00874-0.

## Background

Major depressive disorder (MDD) is among the most prevalent mental health conditions worldwide, affecting approximately 280 million people [1]. This complex and multifactorial disorder arises from the interplay of psychosocial and biological factors. Among the key biological disruptions identified in both MDD patients and animal models of chronic stress are monoamine imbalances, hypothalamic‒pituitary‒adrenal (HPA) axis dysregulation [2, 3], and low-grade systemic inflammation leading to neuroinflammation [4–6]. These alterations have been extensively correlated with depressive symptomatology and behavior, providing critical insights into the biological basis of the disorder.

Psychosocial stressors, such as perceived loneliness, are well-recognized risk factors for depression. Social isolation, which is widely used as a chronic stress paradigm in rodent models, induces depressive-like behaviors and offers a straightforward yet robust model for studying the biological mechanisms of stress. Notably, social isolation impairs behavior in mice [7, 8] and triggers neuroendocrine, immune, and nervous system alterations, including increased levels of proinflammatory cytokines [9, 10].

Despite significant advances, a critical gap remains in understanding how chronic stress differentially affects behavior and biological systems in females. Sexual dimorphism has been documented across multiple domains, including neural circuitry, the gut microbiota, microglial reactivity, peripheral immune responses, and endocrine regulation [11–14]. Moreover, although women exhibit a greater prevalence of MDD than men do, females remain historically underrepresented in both clinical and preclinical research [15, 16]. This underrepresentation limits the development of sex-specific therapeutic strategies and highlights the urgent need for further exploration.

Particularly, in the stress model of social isolation, we have found limited studies addressing depressive and anxiety-like behavior in adult females. Some of them do not observe behavioral changes after four and five weeks of isolation [17, 18], while another one detects depressive-like behavior by the tail suspension test (TST) after eight weeks of isolation [19]. In this study, we examined the effects of chronic social isolation on depressive-like behavior, systemic immune responses, and hippocampal neuroinflammation in both male and female mice, extending the isolation period to seven weeks to determine whether prolonged exposure is required to uncover sex-specific vulnerabilities. Our findings revealed that chronic social isolation impacts body weight, HPA axis activation, and neuroinflammatory markers differently between males and females. While both sexes were affected by social isolation, females exhibited increased corticosterone levels, IL-1β expression, and microglial total area in the hippocampus, compared to males. Additionally, at a baseline level (independent of social isolation stress), we detected differences between males and females in the frequency of CD4 + T cells in the spleen, cytokine expression in the spleen and hippocampus, and astrocytes number.

These findings provide novel insights into sex-specific biological mechanisms underlying stress and depression, underscoring the importance of study duration and sex as critical variables in neuropsychiatric research.

## Methods

### Animals

Adult (11–12-week-old) female and male C57BL/6 mice were used in this study. The animals were housed at the CREAV facility (Universidad de Concepción, Chile) under a 12 h light/dark cycle with food (Lab Diet, 5P00 Prolab RMH 3000, Purina Mills, St. Louis, MO) and water provided *ad libitum.* The experimental group was subjected to eight weeks of social isolation and housed individually in a separate room, but under the same light and temperature conditions as the control group, which was maintained under standard group housing conditions (3–5 animals per cage). All the mice were housed in polycarbonate cages (13 × 19 × 25 cm) with cob bedding, cardboard cylinders, and shredded paper for nesting and environmental enrichment. Olfactory, visual, and auditory contact between the cages was not restricted; however, social interaction with the experimenter was limited to weekly cage cleaning, biweekly body weight monitoring and health checks. All animal procedures followed the guidelines of the Chilean National Research Agency (ANID) for animal experimentation, the ARRIVE guidelines and the US National Research Council’s Guide for the Care and Use of Laboratory Animals. The experimental procedures were approved by the Ethics Committee of Universidad San Sebastián (internal code 03–2019-20) and Universidad de Concepción (internal code: CEBB533-2019; CEBB1114-2022; CEBB 1685–2024).

**Fig. 1:**
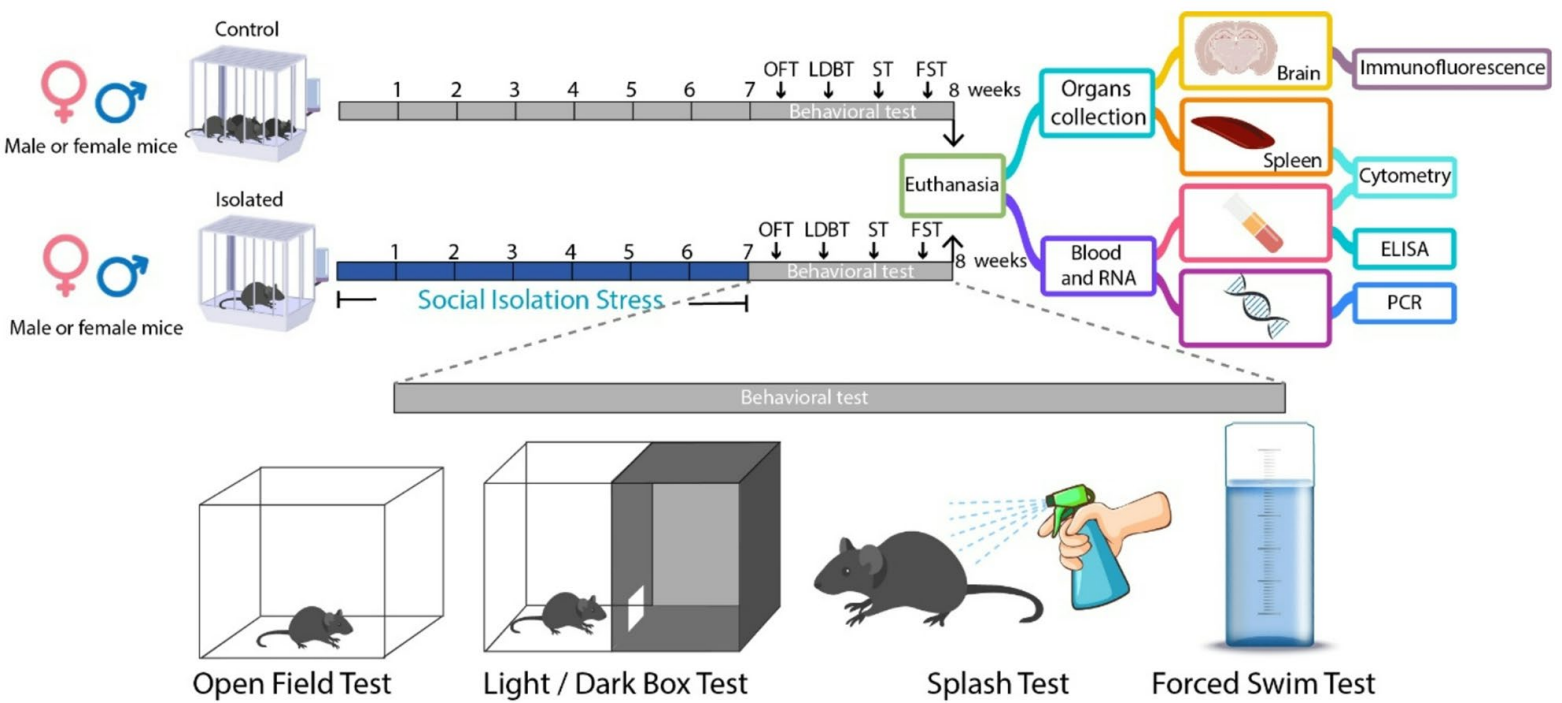
Schematic representation of the social isolation protocol for behavioral analysis and tissue collection

Body weight and food consumption were monitored during the stress protocols, and anxiety- and depressive-like behaviors were analyzed after four and seven weeks of isolation. All animals were tested for behavior prior to tissue collection. Blood samples were obtained before euthanasia at week eight to determine changes in immune cell populations, cytokine levels and corticosterone concentrations. Hippocampal tissue was obtained from a subset of the animals for RNA extraction, whereas the other samples were perfused with 4% paraformaldehyde (PFA) for whole-brain removal and sectioning for posterior immunofluorescence analysis different subsets of animals were used for histology and for qRT-PCR, as the same tissue cannot be processed for both purposes (Fig. 1).

### Behavioral analyses

Behavioral tests were conducted after seven weeks of the protocol to evaluate depressive- and anxiety-like behaviors. The mice were acclimated to the behavioral testing room for 1 h before each test. Behavioral tests were conducted during the animals’ inactive light phase, approximately eight hours into the light cycle. The tests were conducted in the following order: open field test (OFT), light/dark box test (LDBT), splash test (ST), and forced swim test (FST), with a 48-hour interval between each session. The final test was performed two days before euthanasia.

### Open field test (OFT)

The mice were placed in a corner of a square open field box (45 × 45 cm) and allowed to explore freely for 10 min. Activity was recorded, and the distance traveled and average velocity were quantified via ToxTrac software (version 2.91, Umeå University) [20].

### Light/dark box test (LDBT)

The LDBT assesses conflict-avoidance behavior, in which reduced time spent in the illuminated compartment indicates anxiety-like behavior [21]. The apparatus consisted of two equal-sized acrylic chambers (42 × 21 cm), one brightly lit and open and the other dark and enclosed, connected by a 7 × 7 cm doorway. This design contrasts an aversive, exposed environment with a protective, enclosed one. The mice were placed in a corner of the light compartment, and behavior was recorded for 5 min. The time spent in the light area was analyzed as previously described by Moss et al. [22].

### Splash test (ST)

To assess depressive-like behavior related to self-care, a 10% sucrose solution was sprayed onto the dorsal coat of each mouse to induce grooming [23]. The latency to grooming and total grooming time were recorded over 5 min. Decreased grooming behavior was considered indicative of depressive-like behavior.

### Forced swim test (FST)

The FST was used to evaluate behavioral despair. The mice were placed in a transparent acrylic cylinder filled to three-quarters of its volume with water maintained at 22 °C. After an initial 2-minute habituation period (not analyzed), behavior was recorded for 4 min. The immobility time, which represents passive coping or despair, was manually scored via Kinoscope software (version 3.0.4).

### Welfare monitoring, body weight, and food intake

Body weight was measured twice per week as part of welfare monitoring, which also included inspections for potential fight-related injuries. No such injuries were observed; however, this procedure was performed routinely in case aggression occurred. Food intake was estimated by weighing the food offered and remaining after 72 h. Daily food intake per animal was calculated as (food offered − food remaining)/(number of animals × 3 days). Apparent intake values are reported, as we did not correct for food spillage.

### Tissue collection and analysis

At the end of the eight-week protocol, 200–300 µL of blood was collected from the facial vein into tubes containing 20 µL of heparin. A total of 50 µL was used for flow cytometry, and the remaining sample was centrifuged (3,000 rpm, 15 min) to collect the plasma for ELISA.

The mice were anesthetized with ketamine and xylazine, followed by euthanasia via cervical dislocation. Spleens were harvested and placed in 1X PBS supplemented with 5% fetal bovine serum (FBS). The brains were carefully removed, and the hippocampi were dissected using a mouse brain slicer matrix for 1 mm coronal sections (RWD LifeScience, China) and stored in TRIzol reagent for mRNA analysis. For immunofluorescence analysis, the animals were transcardially perfused with 1X PBS followed by 4% PFA in PBS. Following decapitation, the brains were collected and fixed in 4% PFA, at 4 °C for 48 h. For some mice, adipose tissue was postfixed in 4% PFA at 4 °C for 48 h.

### ELISA

Plasma levels of corticosterone, interleukin (IL)−6 and transforming growth factor (TGF)-β were quantified via commercial ELISA kits according to the manufacturer’s instructions: Corticosterone (cat. no. KGE009, R&D Systems, Minneapolis, USA), IL-6 (cat. no. 431304, Biolegend, San Diego, USA), and TGF-β1 (cat. no. 88–8350-22, Thermo Fisher, California, USA).

### Flow cytometry analysis

Peripheral blood mononuclear cells (PBMCs) and splenocytes were isolated from whole blood and spleen, respectively. The blood was treated with ACK lysis buffer (Gibco, Life Technologies, Paisley, United Kingdom) at 37 °C for 10 min, whereas the spleens were mechanically dissociated via a cell strainer and treated with ACK buffer for 2 min at room temperature. PBMCs or 2 × 10⁶ splenocytes were stained with anti-CD45-FITC (1:200, BD Biosciences, San Diego, CA, USA) and anti-Ly6G-APC (1:200, BioLegend, San Diego, CA, USA) for neutrophil analysis and with anti-CD4-APC (1:100, BioLegend, San Diego, CA, USA) and anti-CD44-PerCP-Cy5.5 (1:200, BioLegend) for the analysis of effector T lymphocytes. Staining was performed in 1X PBS + 5% FBS (Gibco, Life Technologies, Paisley, UK) at 4 °C for 30 min in the dark.

For intracellular FOXP3 staining, the cells were fixed and permeabilized using the FOXP3/Transcription Factor Staining Buffer Set (eBioscience, Thermo Fisher, Carlsbad, CA, USA) at 4 °C in the dark for 45 min and then incubated with anti-FOXP3-PE (1:200, Invitrogen, Thermo Fisher, Carlsbad, CA, USA) at 4 °C for 30 min in the dark. For Th1/Th17 analysis, 2 × 10⁶ splenocytes were stimulated with 50 ng/mL phorbol 12-myristate 13-acetate (PMA) (Merck, Darmstadt, Germany), 1 µg/mL ionomycin (Merck, Darmstadt, Germany) and 10 µg/mL brefeldin A (Sigma‒Aldrich, St. Louis, MO, USA) at 37 °C for 4 h. After surface staining with anti-CD4-APC antibody (1:200, BD Pharmingen) at 4 °C for 30 min, the cells were permeabilized for 45 min at 4 °C using the FOXP3 Cytofix/Cytoperm Buffer Kit (eBioscience, USA) and incubated overnight (16 h) at 4 °C with fluorophore-conjugated antibodies against IFN-γ-PerCP-Cy5.5 (1:100, BD Pharmingen) and IL-17 A-AF488 (1:100, BD Pharmingen) to identify Th1 and Th17 lymphocytes, respectively. The samples were washed, resuspended in 1X PBS, and stored at 4 °C until analysis on a FACSCanto II flow cytometer (BD Biosciences, San Diego, CA, USA). The data were analyzed via FlowJo software, version 10.6 (Tree Star).

### Gene expression analysis

Total RNA was extracted from the spleen and hippocampus using TRIzol (Invitrogen, Thermo Fisher, Carlsbad, CA, USA) following the manufacturer’s protocol. The RNA concentration and purity were assessed using a UV‒Vis EPOCH microplate spectrophotometer (BioTek Instruments Inc., Winooski, VT, USA) at 260/280 nm. The RNA was subjected to DNase I treatment (Sigma‒Aldrich, St. Louis, MO, USA) to eliminate contaminating genomic DNA, and 2 µg of total RNA was reverse transcribed to cDNA using the iScript cDNA Synthesis Kit (Bio-Rad, Hercules, CA, USA). qRT-PCR was performed using SYBR Green SsoAdvanced Universal Supermix (Bio-Rad, Hercules, CA, USA) on a QuantStudio 3 Real-Time PCR Detection System (Applied Biosystems, Thermo Fisher Scientific) under the following conditions: initial denaturation at 95 °C, followed by 40 cycles of 55 °C for 30 s and 72 °C for 30 s. Primer sequences for target genes (e.g., TNF-α, IL-6, IL-1β, TGF-β, IL-10, FOXP3, IFN-γ, IDO, C3, CD68, CD86, CD206, arginase-1, and IL-4) and the reference gene (18 S) are listed in Supplementary Table 1. Fold changes were calculated using the 2^-ΔΔCt method and normalized against 18 S; and the average ΔCt was obtained from the geometric means of non-stressed females and males combined. The values from independent biological experiments were adjusted using a correction factor based on the mean ΔCT of control animals from a reference experiment including all experimental groups.

### Histological processing and staining of adipose tissue

At the end of behavioral testing, adipose tissue samples were collected and dehydrated through a graded ethanol series (70%, 96%, and 99.5%) (Winkler, Chile) before being embedded in histological-grade paraffin (Leica) at 60 °C to form blocks. Once cooled, the paraffin blocks were sectioned at a thickness of 7 μm via a microtome (RM2125RT, Leica) and mounted on glass slides (LaborGläser). For staining, the sections were deparaffinized with xylol and rehydrated through a descending ethanol series (99.5%, 96%, and 70%), followed by the addition of distilled water. Hematoxylin‒eosin (H&E) staining was performed using Harris hematoxylin (Sigma‒Aldrich, St. Louis, MO, USA) and eosin (Sigma‒Aldrich, St. Louis, MO, USA). Finally, the sections were dehydrated and mounted with Entellan™ (Sigma‒Aldrich, St. Louis, MO, USA).

### Brain histological processing and immunofluorescence

Following perfusion with 4% PFA, the brains were postfixed in the same fixative for 48 h at 4 °C. Tissues were cryoprotected by immersion in 30% sucrose (Merck, Darmstadt, Germany) in PBS at 4 °C for 7 days, embedded in Tissue-Tek optimal cutting temperature (OCT) compound (TissueTek; Sakura Finetek USA), and sectioned at 30 μm using a cryostat (RWD minux FS800). The sections were mounted on slides (LaborGläser) and stored at −20 °C until further processing. Free-floating 30 μm sections were incubated in Tris phosphate buffer (84 mM Na_2_HPO_4_, 35 mM KH_2_PO_4_, 120 mM NaCl, and 10 mM Tris, pH 7.8), permeabilized with 0.3% Triton-X-100 in Tris phosphate buffer and blocked with 3% BSA in Tris phosphate buffer. The following primary and secondary antibodies were used: rabbit anti-iba1 antibody (1:400, Wako, Fujifilm), mouse anti-GFAP antibody (1:200, Sigma‒Aldrich, Merck), AlexaFluor 488-conjugated goat anti-rabbit (1:200, Invitrogen, Thermo Fisher, Carlsbad, CA, USA), and Alexa Fluor 594-conjugated donkey anti-mouse (1:200, Invitrogen, Thermo Fisher, Carlsbad, CA, USA). Nuclei were labeled with TOPRO-3 (1:750, Invitrogen, Thermo Fisher, Carlsbad, CA, USA). Primary antibodies were diluted in Tris phosphate buffer containing 0.2% Triton-X and 1% BSA and incubated overnight at 4 °C. The secondary antibodies were diluted in Tris phosphate buffer with 1% BSA for 2 h at room temperature.

Coverslips were mounted using DAKO fluorescence mounting medium (Agilent Technologies). Micrographs at 100x and 400x magnification were acquired using a LSM700 confocal microscope (Zeiss) at the CMA Bio-Bio facility.

### Image processing and morphometric analyses

The quantification of astrocytes and microglia, as well as Sholl and skeleton analyses of microglia, was performed to evaluate morphological and structural changes in the dentate gyrus (DG) of the hippocampus, specifically in the region below the hippocampal fissure, which includes both the granule and molecular layers. We analyzed the dorsal hippocampus, which receives afferences from hypothalamus and amygdala, and has been analyzed in other studies of chronic stress and neuroinflammation, finding differences in microglia number and neurogenesis [7, 24, 25].

Skeleton analyses were conducted to assess Iba-1-positive cell morphology via FIJI/ImageJ software. Briefly, 400x images of Iba-1-labeled microglia were imported into FIJI. For each animal, eight Iba-1-positive cells with representative morphologies were randomly selected in a blinded manner. The images were binarized, and morphological parameters such as soma area and convex hull area were quantified using the measurement tool in ImageJ. Branching analyses were performed on skeletonized binary images using the AnalyzeSkeleton plugin (http://imagejdocu.tudor.lu/*).* Additionally, Sholl analyses were conducted using the SNT FIJI plugin [26], with the starting radius, step size, and ending radius set at 5 μm, 5 μm, and 100 μm, respectively. The number of branch intersections with each concentric circle was recorded.

### Statistical analysis

The data are presented as the means ± standard deviations (SDs). Before analysis, outliers were identified and removed using the robust regression and outlier removal (ROUT) method (Q = 2%). Normal distribution was assessed via the Shapiro‒Wilk test. Two-way ANOVA was performed to assess the effects of sex and stress. When significant main effects or interactions were detected, the Bonferroni post-hoc correction was applied for multiple comparisons. Statistical analyses were performed via GraphPad Prism version 8.4 (GraphPad Software Inc., La Jolla, CA, USA). The results were considered statistically significant at *p* < 0.05.

## Results

### Prolonged isolation stress induces depressive-like behavior in male and female mice

The few studies that have analyzed female mice in the social isolation paradigm have indicated that after four weeks of isolation, only males display depressive- and anxiety-like behaviors, suggesting that females may be resistant to this type of stress [17, 18]. However, longer exposure times have not been thoroughly assessed to determine whether this resistance is genuine or simply reflects a delayed onset of behavioral alterations. Here, male and female mice were subjected to a social isolation protocol for five or eight weeks, with behavioral assessments performed at week four and seven, respectively (Fig. 1).

**Fig. 2:**
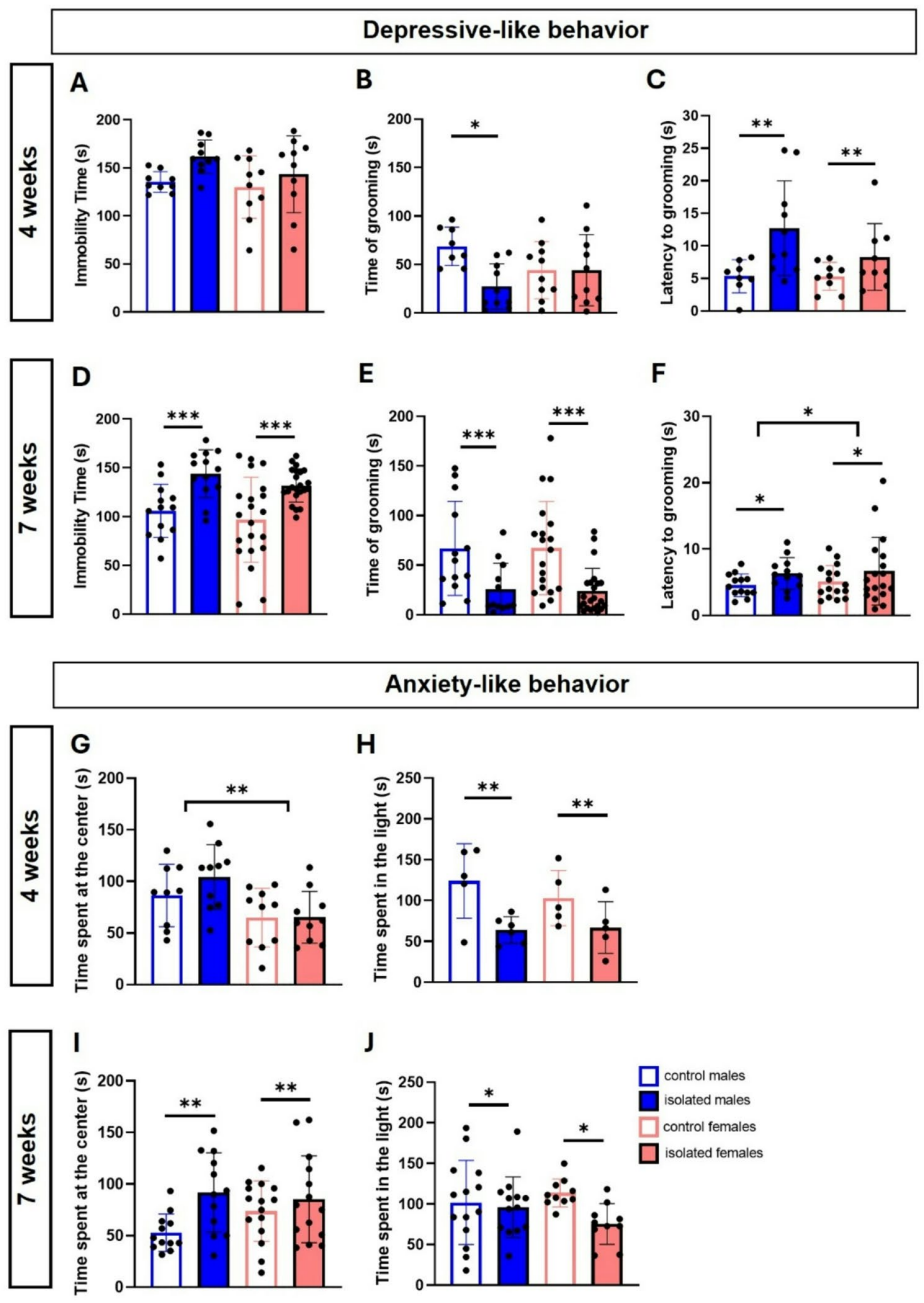
Effects of social isolation on depressive and anxiety-like behavior. Total immobility time during the forced swim test after 4 **(A)** and 7 weeks **(D)**, time spent grooming during the splash test after 4 **(B)** and 7 weeks **(E)**, and latency to grooming during the splash test after 4 **(C)** and 7 weeks **(F)**. In addition, anxiety-like behavior was analyzed by measuring the time spent at the center of the arena in the open field test after 4 **(G)** and 7 weeks **(I)**, and the time spent in the illuminated area of the box during the light‒dark box test after 4 **(H)** and 7 weeks **(J)**. *p < 0.05, **p < 0.01, ***p < 0.001, two-way ANOVA followed by Bonferroni post-hoc correction. For the 4-week isolation protocol, nine males and ten female mice per group were used from two independent experiments (except for the LDBT, which was not performed in both cohorts; for that reason, only five males and females were used from one experiment). For the 7-week isolation protocol, thirteen male mice were used per group from three independent experiments, and nineteen female mice per group were used from four independent experiments (except for anxiety-like behavior, where *n* = 10 females from three experiments were used per group)

After four weeks of social isolation, we did not find changes in the FST (Fig. 2A) by two-way ANOVA. However, we found a significant interaction between sex and isolation on the grooming time in the splash test (F (1, 34) = 4.942, *p* < 0.033); post-hoc comparisons showed that isolated males spend significantly less time grooming than control males (*p* = 0.0263) (Fig. 2B). In the same test, a significant effect of stress was observed for the latency of grooming, with isolated mice showing longer latency to start grooming (F (1, 32) = 9.951, *p* = 0.0035) (Fig. 2C). After seven weeks of isolation, on the other hand, two-way ANOVA analyses revealed a significant main effect of isolation in the FST (F (1, 50) = 16.90, *p* = 0.0001) with socially isolated mice showing higher immobility during the test (Fig. 2D). Social isolation also reduced total grooming time in the ST (F (1, 49) = 12.3, *p* < 0.0001) (Fig. 2E) and increased grooming latency (F (1, 48) = 8.784, *p* = 0.047). In addition, grooming latency showed sex-related differences (F (1, 48) = 4.655, *p* = 0.036) (Fig. 2F).

Regarding anxiety-like behavior, a significant main effect of sex was observed after four weeks of social isolation in the OFT, with females in general spending less time at the center of the arena, (F (1, 35) = 10.68, *p* = 0.0024) (Fig. 2G), while a main effect of isolation was observed after seven weeks (F (1, 49) = 7.483, *p* = 0.0087). But unexpectedly, isolated mice spend more time at the center, compared to controls, suggesting increased exploratory behavior (Fig. 2I). For the LDBT, on the other hand, a main effect was observed for isolation after four (F (1, 17) = 11.23, *p* = 0.0038) and seven weeks (F (1, 40) = 6,590, *p* = 0,0141), showing decreased time in the illuminated side of the box (Fig. 2G, H).

Overall, our results show that social isolation stress can promote depressive-like behavior in both sexes, but females need more time to develop more robust behavioral changes. We continued to analyze biological changes only in the cohort exposed to social isolation for seven weeks.

### Isolation stress alters body weight and adipose tissue differently in males and females

**Fig. 3:**
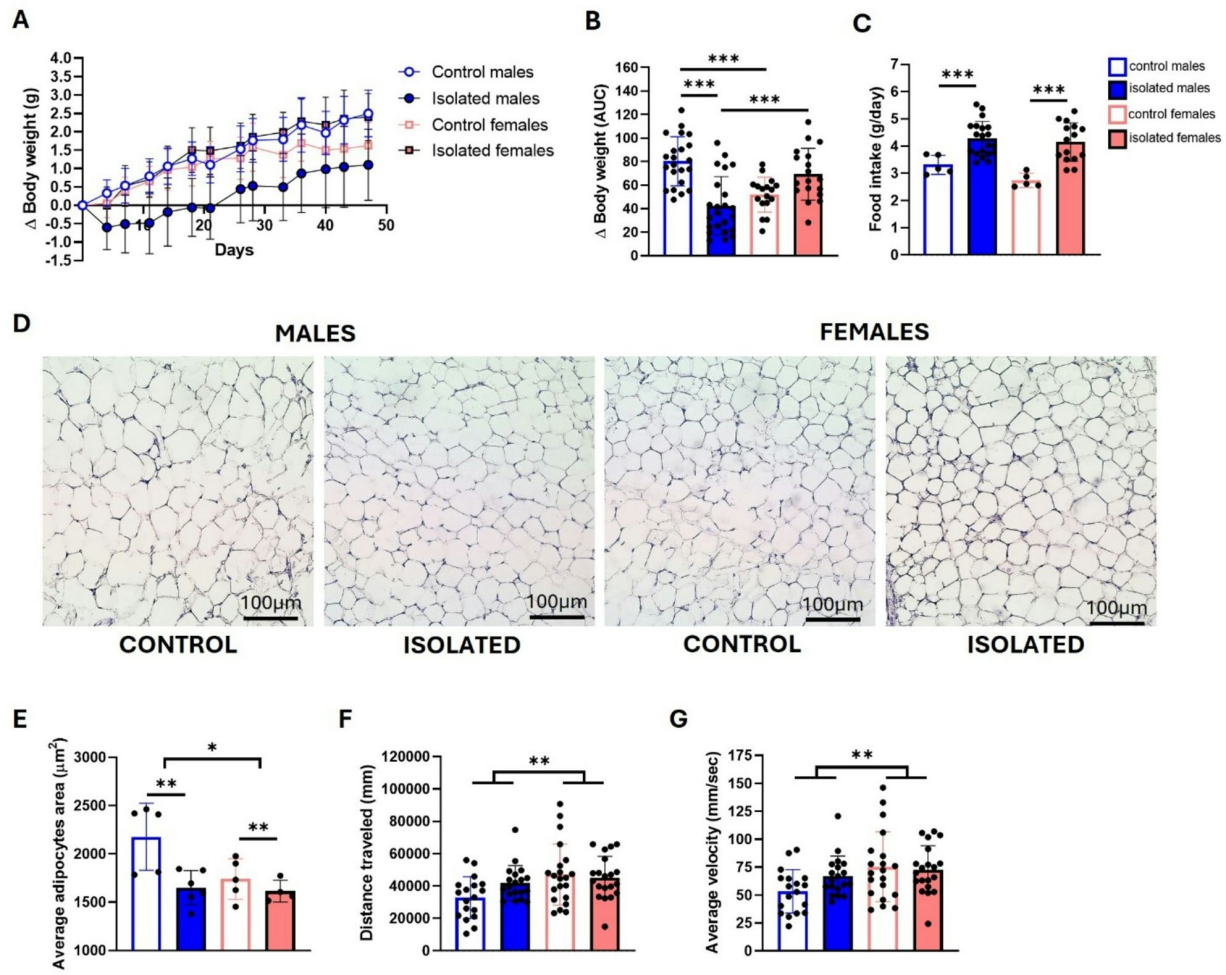
Analysis of body weight gain, fat mass, and locomotion in socially isolated mice. Delta body weights for control and isolated mice **(A)**, area under the curve analysis **(B)**, and daily food intake **(C).** Representative histological images of adipose tissue from control and isolated mice **(D)**, with their corresponding quantification of the average adipocyte area in µm^2^ **(E)**. Scale bars: 100 μm. Locomotion analysis in the open field test was recorded, and the distance traveled **(F)** and average velocity **(G)** were measured. *p < 0.05, **p < 0.01, ***p < 0.001; two-way ANOVA followed by Bonferroni post-hoc correction. For body weight, food intake, and locomotion analysis, twenty-three male mice from three independent experiments and twenty-one female mice from four independent experiments were used per group. For adipose tissue analysis, five controls per sex were used: six isolated males and four isolated females

Body weight was measured twice a week to monitor animal welfare, given that various chronic stress paradigms induce weight loss in male mice [27, 28]. Delta body weight curves revealed that isolated males lost weight during the first weeks and subsequently gained significantly less weight than controls did, whereas isolated females did not lose weight (Fig. 3A). This can also be observed in the area under the curve (AUC) plots, where two-way ANOVA revealed a significant interaction between sex and isolation (F (1, 76) = 33.06, *p* < 0.0001), indicating that the impact of isolation on body weight differed between males and females. Post-hoc comparisons showed that isolated males had significantly lower body weight than control males (*p* < 0.0001). In addition, control males differed from control females (*p* = 0.0006), and isolated males also differed from isolated females (*p* = 0.009), indicating that both isolation and sex contributed distinctly to the observed body-weight trajectories (Fig. 3B). Interestingly, the lower body weight in isolated males was not attributable to reduced food intake. In fact, isolated animals consumed significantly more food than controls, as confirmed by a main effect of isolation in the ANOVA two-way analysis (F (1, 47) = 6.486, *p* = 0.0142) (Fig. 3C).

Histological analysis of adipose tissue revealed significant effects of social isolation (F (1, 17) = 9.195, *p* = 0.0075) and sex (F (1, 17) = 6.105, *p* = 0.0244), indicating overall differences in adipocyte size across groups (Fig. 3D–E). Although the two-way ANOVA did not show an interaction, a trend suggests that the reduction in adipocyte size was more pronounced in isolated males than in isolated females. This visual pattern is consistent with the stronger body-weight effects observed in males, even though it cannot be statistically attributed to an interaction based on the present analysis.

Locomotor activity was obtained with OFT after seven weeks of isolation. Our results revealed no isolation effects; however, significant sex-related differences were observed in total distance traveled (F (1, 74) = 7.341, *p* = 0.0084) and average velocity (F (1, 74) = 7.030, *p* = 0.0098), with females reaching higher values (Fig. 3F, G).

### Social isolation stress increases neutrophil frequency and downregulates Th1 and Th17 cytokines expression

**Fig. 4:**
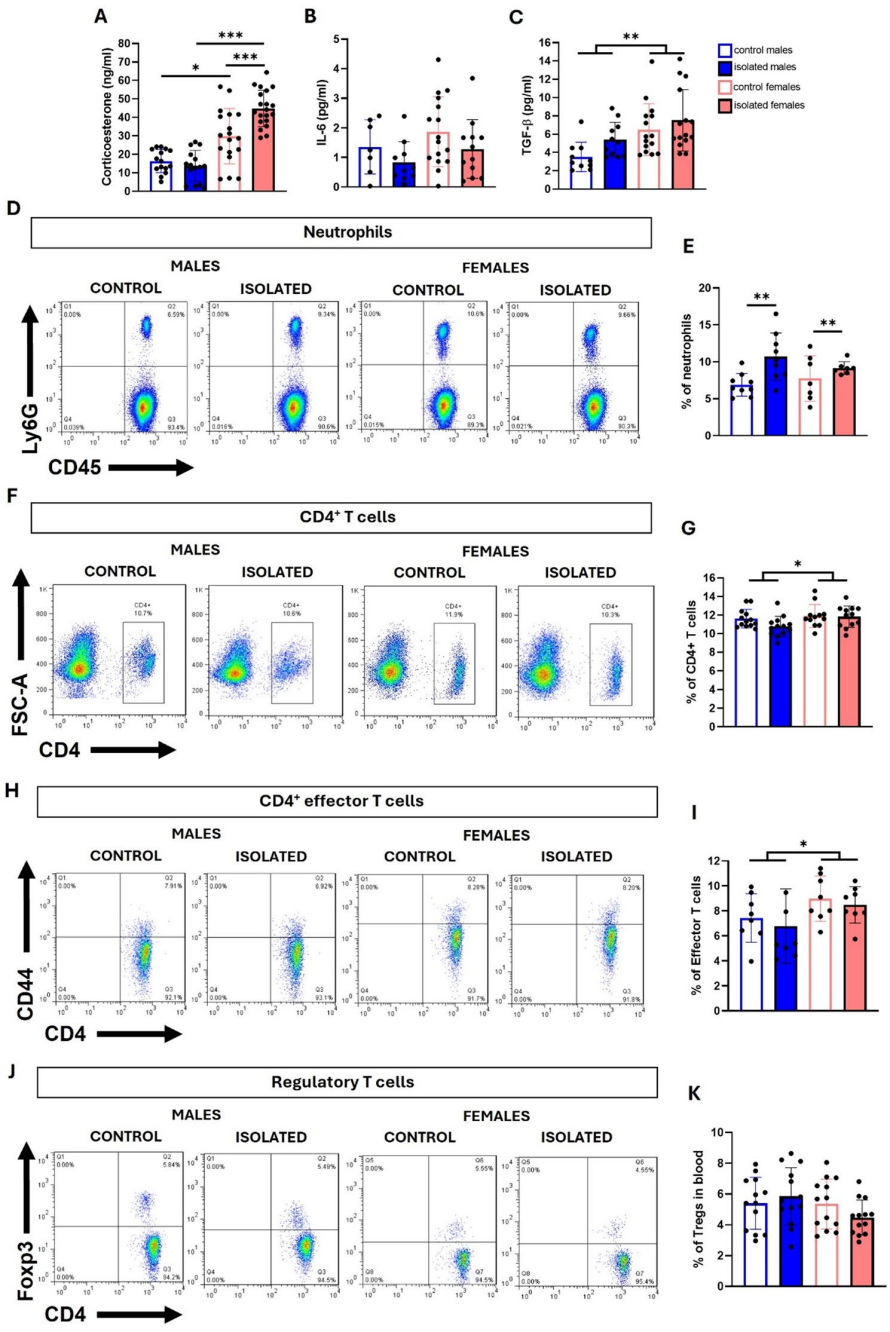
Inflammatory biomarkers in blood from socially isolated mice. Plasma levels of corticosterone **(A)**, IL-6 **(B)**, and TGF-β **(C)** were measured via ELISA. Flow cytometry plots and their respective quantification bar graphs for neutrophils **(D**,** E)**, total CD4^+^ T cells **(F**,** G)**, CD4^+^ effector T cells **(H**,** I)**, and regulatory T cells **(J**,** K)** in blood. *p < 0.05, **p < 0.01, ***p < 0.001; two-way ANOVA followed by Bonferroni post-hoc correction. Blood was extracted from all the mice that underwent behavioral analysis, but sufficient plasma could not be recovered from all the samples for the three ELISAs, which explains the disparity in n values. For flow cytometry analysis, thirteen samples from male and female mice from three independent experiments were used per group

Corticosterone levels are frequently used as a measure of the stress response [29]. We determined the basal plasma levels of corticosterone through ELISA in blood samples collected two to three days after performing the last behavioral test (i.e., the FST). Two-way ANOVA revealed significant main effects of sex (F(1, 65) = 63.8, *p* < 0.0001) and social isolation (F(1, 65) = 6.673, *p* = 0.0120), as well as a significant sex × isolation interaction (F(1, 65) = 12.79, *p* = 0.0007). Post-hoc comparisons showed significant differences between control and isolated females (*p* < 0.0001), between isolated males and females (*p* < 0.0001), and between control males and females (*p* = 0,017) (Fig. 4A).

Because chronic stress is known to alter the immune system and promote low-grade inflammation, we analyzed the presence of pro- and anti-inflammatory cytokines in plasma, as well as circulating immune cell populations in blood. We did not detect changes in IL-6 (Fig. 4B). TGF-β levels were influenced by sex (F (1, 47) = 11.45, *p* = 0.0015), with higher levels in females than in males (Fig. 4C).

We evaluated whether social isolation induced changes in neutrophils, as they have been detected to increase in the circulation of MDD patients, showing an elevated neutrophil to lymphocyte ratio (NLRs) [30]. Flow cytometry revealed significant effects of social isolation (F (1, 28) = 9.371, *p* = 0.0048).

Total CD4^+^ T cells and CD4^+^ subpopulations were also analyzed on the basis of previous reports showing that T cells are also impaired in chronic stress models and that CD4^+^ T cells are indeed required for the development of anxiety [31]. No stress-related differences were detected in the total frequency of CD4^+^T cells or effector T cells (CD4^+^ CD44^hi^) (Fig. 4F - I); however, sex had a significant effect on total CD4⁺ T cells (F(1, 46) = 4.443, *p* = 0.0405) and on effector T cells (F(1, 28) = 4.649, *p* = 0.0398), with higher number on females.

Previous studies have shown that some models of stress exhibit a reduced frequency of regulatory T cells (Tregs) [27, 32], a cell population key to maintaining immune homeostasis. We aimed to analyze whether social isolation induces changes in Tregs frequency, but we did not detect differences between the control groups and individuals isolated from either male or female mice (Fig. 4J, K).

**Fig. 5:**
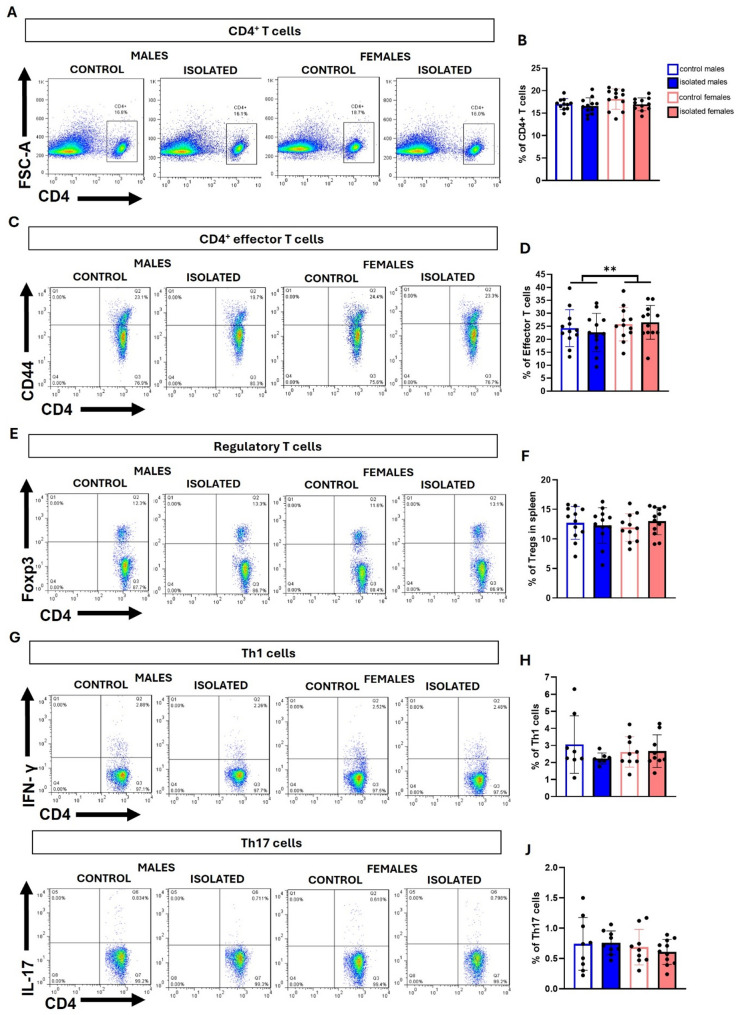
Flow cytometry analysis of inflammatory biomarkers in spleens from socially isolated mice. Flow cytometry plots and their respective quantification bar graphs for total CD4^+^ T cells **(A**,** B)**, CD4 + effector T cells **(C**,** D)**, regulatory T cells **(E**,** F)**, Th1 cells **(G**,** H)**, and Th17 cells **(I**,** J)** in the spleen. *p < 0.05, **p < 0.01, ***p < 0.001; two-way ANOVA followed by Bonferroni post-hoc correction. Twelve spleens from male and female mice from three independent experiments (apart from Th1 and Th17 analyses, where *n* = 9 per group) were used per group

**Fig. 6:**
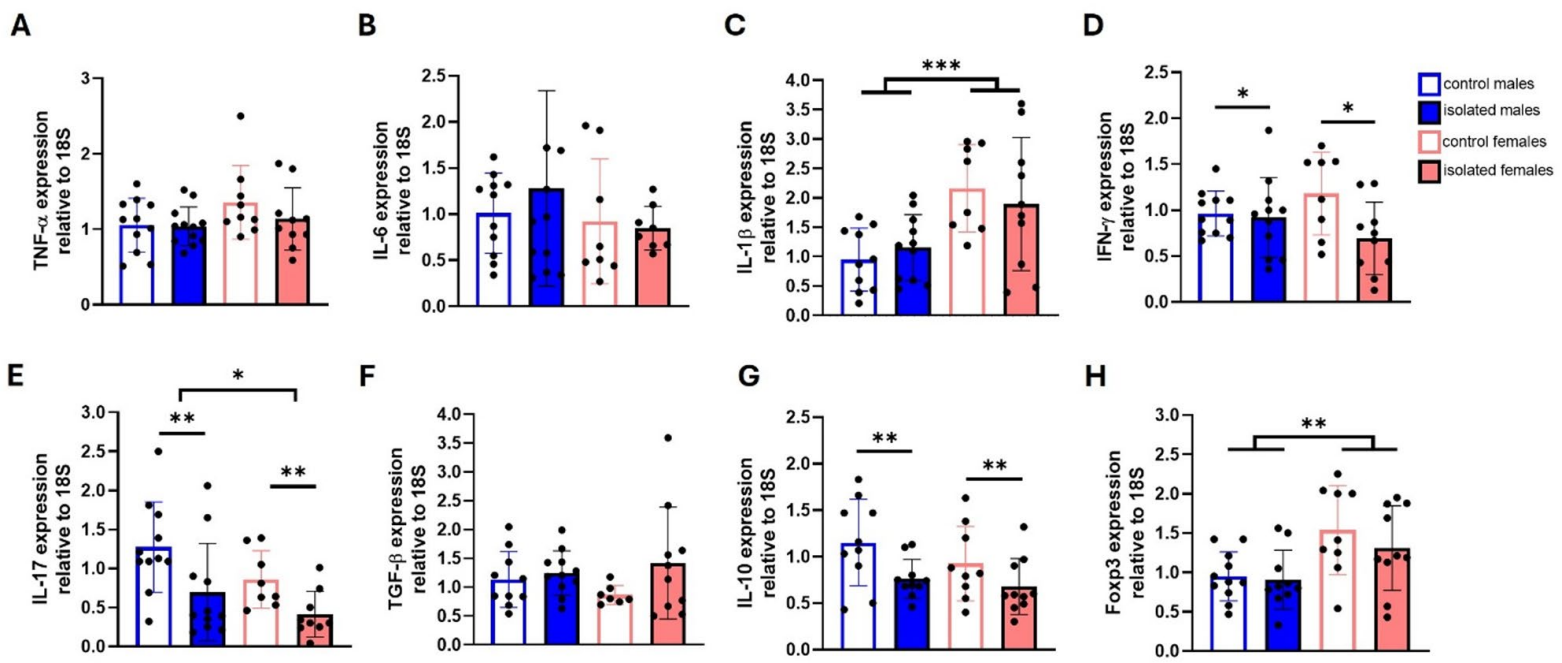
Cytokine expression analysis of the spleens of control and isolated mice. qRT-PCR analysis of the expression of the cytokines TNF-α **(A)**, IL-6 **(B)**, IL-1β **(C)**, IFN-γ **(D)**, IL-17 **(E)**, TGF-β **(F)**, and IL-10 **(G)** and the Tregs cell transcription factor Foxp3 **(H).** *p < 0.05, **p < 0.01, ***p < 0.001; two-way ANOVA followed by Bonferroni post-hoc correction. Twelve spleens from male and ten from female mice from three independent experiments were used per group

To gain better insight into the possible inflammatory state, we analyzed T cell populations and cytokines in the spleen. Analysis of T cell populations revealed no isolation-induced changes in total CD4^+^ T cells (Fig. 5A, B), effector T cells (Fig. 5C, D), Tregs cells (Fig. 5E, F), Th1 (Fig. 5G, H), or Th17 populations (Fig. 5I, J). However, consistent with the findings in blood, sex had a significant effect on effector T cells (F (1, 47) = 6.71 *p* = 0.0127). qRT-PCR revealed no significant differences in the expression of the cytokines TNF-α, IL-6, and TGF-β (Fig. 6A, B, F). In contrast, significant sex effects were detected for IL-1β (F (1, 35) = 15.08, *p* = 0.0004) and Foxp3 (F (1, 37) = 4.573, *p* = 0.0392), with females having higher expression (Fig. 6C, H). A main effect of isolation was observed for INF-γ (F (1, 36) = 4.709, *p* = 0.0367) and IL-10 (F (1, 35) = 7.833, *p* = 0.0083), with lower expression in isolated mice (Fig. 6D, G). Finally, IL-17 expression showed both a significant sex effect (F (1, 35) = 4.603, *p* = 0.0389) and an isolated-related effect (F (1, 35) = 9.841, *p* = 0.0035), with lower expression in isolated mice and in females compared to males (Fig. 6E).

### Social isolation induces neuroinflammation in both sexes, but isolated female mice exhibit increased expression of IL-1β and higher microglia area

**Fig. 7:**
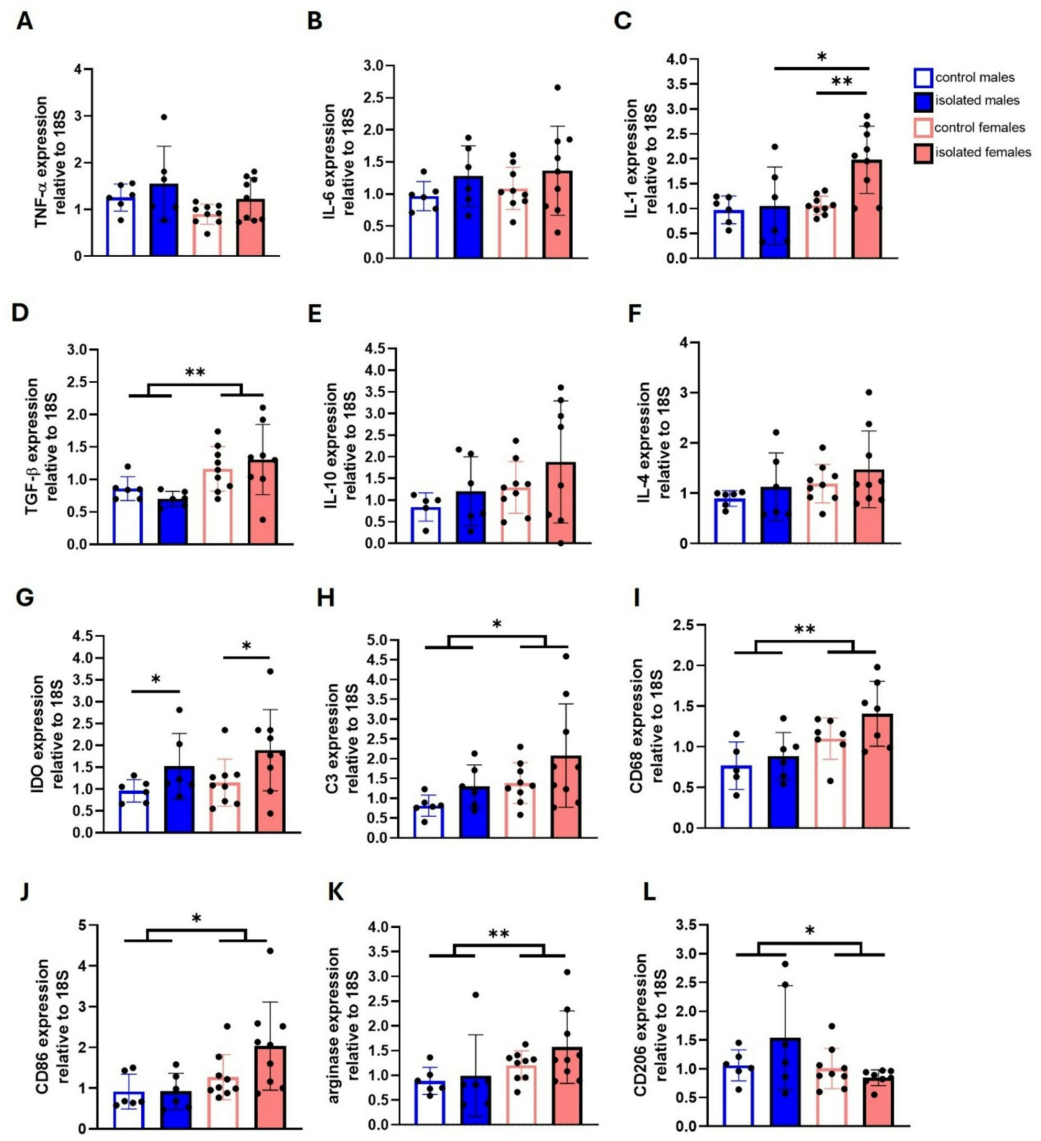
Cytokine expression analysis and microglial reactivity markers in the hippocampi of control and isolated mice. qRT-PCR for TNF-α **(A)**, IL-6 **(B)**, IL-1β **(C)**, TGF-β **(D)**, IL-10 **(E)**, IL-4 **(F)**, IDO **(G)**, C3 **(H)**, CD68 **(I)**, CD86 **(J)**, arginase-1 **(K)** and CD206 **(L) expression.** *p < 0.05, **p < 0.01, ***p < 0.001; two-way ANOVA followed by Bonferroni post-hoc correction. Six brains from males and nine from female mice from two independent experiments were used per group

We next evaluated neuroinflammatory markers in the brain, specifically in the dorsal hippocampus. Cytokine expression and microglial phenotype markers were assessed using qRT-PCR. Two-way ANOVA revealed a significant main effect of sex for the anti-inflammatory cytokine TGF-ꞵ (F (1, 25) = 11.31, *p* = 0.0025), complement component C3 (F (1, 26) = 4.821, *p* = 0.0372) and CD68 (F (1, 21) = 11.11, *p* = 0.0032), the co-stimulatory molecule CD86 (F (1, 26) = 7.305, *p* = 0.0120), and anti-inflammatory microglial markers arginase-1 (F (1, 26) = 4.337, *p* = 0.0473) and CD206 (F (1, 25) = 4.397, *p* = 0.0463) (Fig. 7D, H, I-L). In general, females exhibited higher expression levels compared to males, except for CD206.

The expression of TNF-α, IL-6, IL-10, and IL-4 was not affected by isolation or sex (Fig. 7A, B, E, F). Only IL-1ꞵ expression showed significant effects of both sex and isolation, as well as their interaction (F (1, 26) = 6.542, *p* = 0.0167; F (1, 26) = 6.328, *p* = 0.0184); F (1, 26) = 4.425, *p* = 0.0452). Post-hoc analysis reveals greater expression of IL-1ꞵ was observed in isolated females than in control females (*p* = 0,0046), as well as compared to isolated males (*p* = 0,0113) (Fig. 7C).

Under pathological conditions, microglia can release cytotoxic molecules that disturb neuronal neurotransmission, such as quinolinic acid [33, 34]. This compound is generated by an enzymatic pathway led by the activity of the enzyme indoleamine-2,3-dioxygenase (IDO). For this reason, we also evaluated changes in IDO expression. Two-way ANOVA revealed a significant main effect of social isolation (F (1, 26) = 6.450, *p* = 0.0174) (Fig. 7G).

**Fig. 8:**
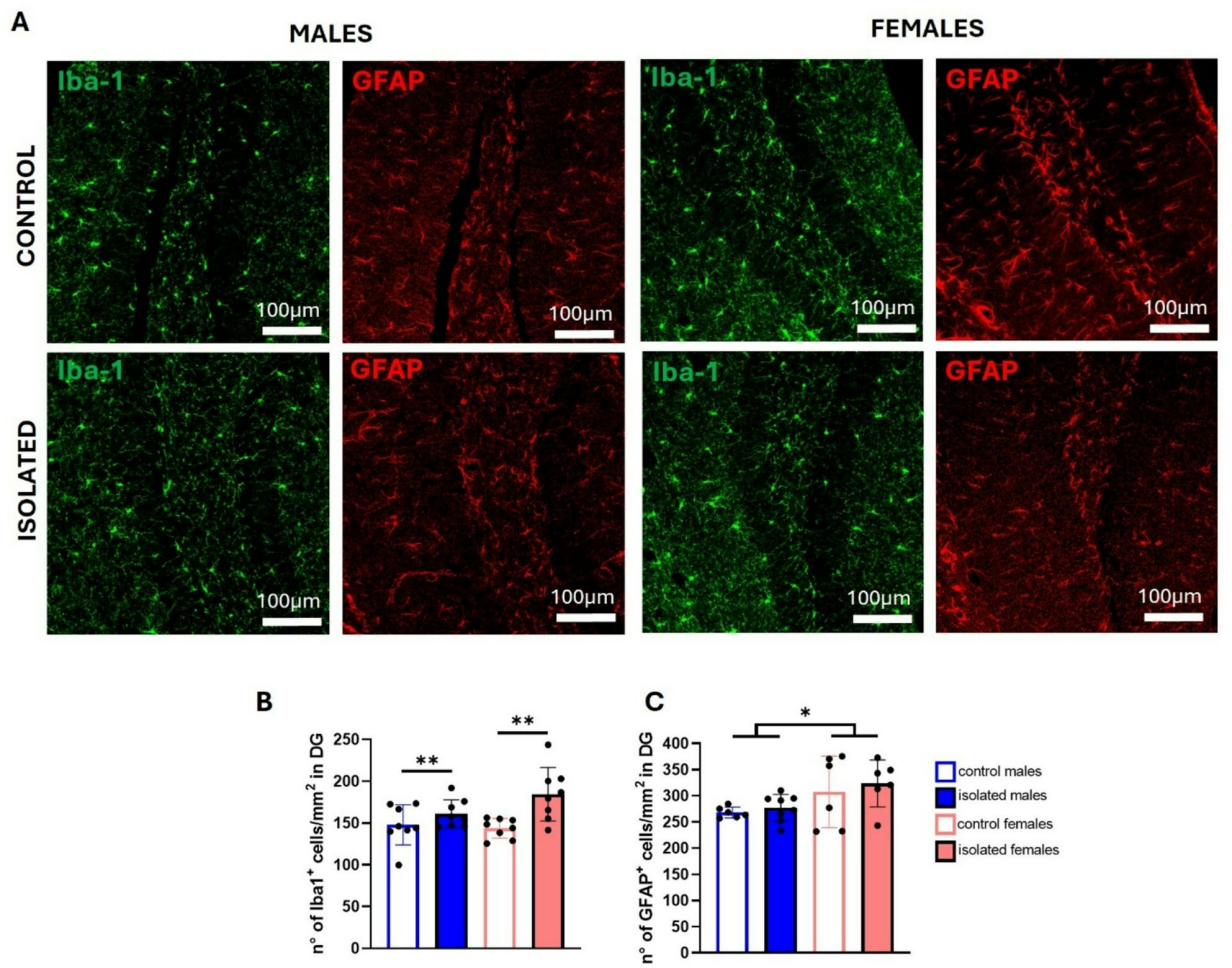
Iba1 and GFAP expression in the hippocampus dentate gyrus. Representative confocal microscopy images of Iba-1 (green) and GFAP (red) immunofluorescence **(A).** Scale bars: 100 μm. Bar graphs for the quantification of Iba-1^+^ **(B)** and GFAP^+^ cells **(C)**. *p < 0.05, **p < 0.01, ***p < 0.001; two-way ANOVA followed by Bonferroni post-hoc correction. Eight brains from two independent experiments were used per sex and group

Several studies have shown that chronic stress induces changes in the number and morphology of microglia and astrocytes [27, 35–38], so we analyzed changes in these cells through immunofluorescence for Iba-1 and GFAP, respectively, using thick cryostat brain sections (Fig. 8A). Two-way ANOVA showed a main effect of isolation stress on microglial number, observing increased number in isolated mice (F (1, 28) = 11.38, *p* = 0.0022) (Fig. 8B), while for astrocytes a significant main effect of sex was observed (F (1, 22) = 6.771, *p* = 0.0163) (Fig. 8C).

**Fig. 9:**
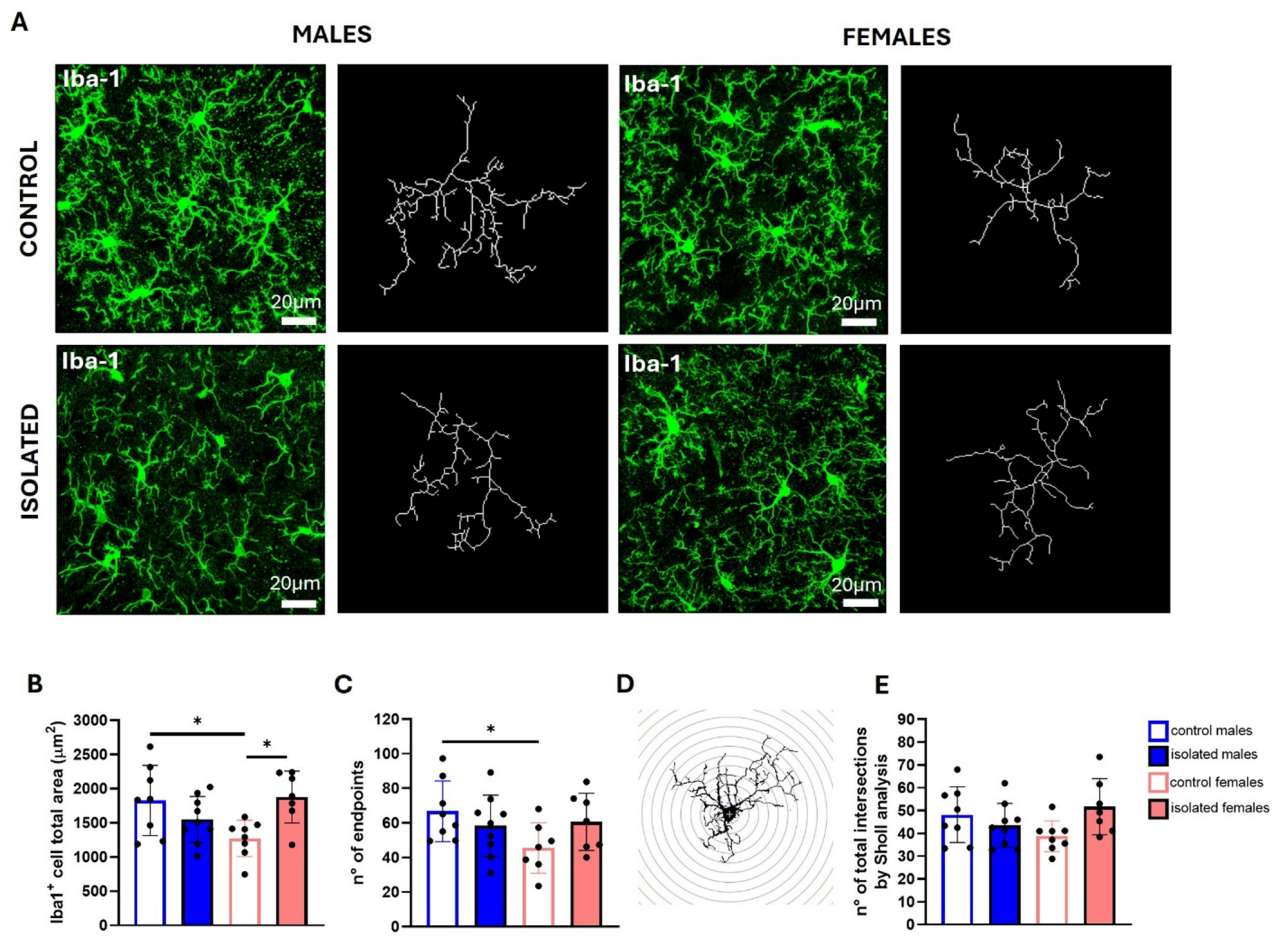
Morphological analysis of microglia in the dentate gyrus. Representative confocal microscopy images of immunofluorescence for Iba-1 (green) using the maximum projection intensity of z-stacks, with representative skeletonization of single microglia for each group of mice (white skeleton on a black background) **(A).** Scale bars: 20 μm. Bar graphs for the quantification of the total area occupied by Iba-1^+^ cells, also known as the convex hull **(B)** and the number of endpoints **(C)**. A representative image from Sholl analysis **(D)** and a bar graph showing the quantification of total intersections via Sholl analysis (**F)** are shown. *p < 0.05, **p < 0.01, ***p < 0.001; two-way ANOVA followed by Bonferroni post-hoc correction. Eight brains from two independent experiments were used per sex and group

A detailed analysis of microglial morphology (Fig. 9A-E) revealed a significant interaction between sex and isolation for the territory occupied by Iba1^+^ cells in the dentate gyrus (F (1, 28) = 10.54, *p* = 0.0030), with isolated females showing a larger occupied area compared with their controls (*p* = 0.0195), and control males exhibiting larger areas than control females (*p* = 0,0289) (Fig. 9B). A significant interaction was also detected for the number of microglial endpoints (F (1, 28) = 4.766, *p* = 0.0376) (Fig. 9C), where under control conditions, males displayed a greater number of endpoints than females (*p* = 0.0445), suggesting that at a basal level, the microglia of females exhibit lower complexity. Similarly, Sholl analysis revealed a significant interaction between sex and isolation (F (1, 28) = 5.740, *p* = 0.0235) (Fig. 9D), although no significant post-hoc differences were found (Fig. 9E).

Although social isolation increased IDO expression in the hippocampus and the number of microglia in both male and female mice, only in females significantly increased IL-1β and microglia total area, suggesting higher reactivity and morphology remodeling.

A comprehensive summary of our results, highlighting the differences observed by sex and stress, is depicted in Table 1.

**Table 1:** Summary of main results observed by sex and stress

Sex-related differences		
Variable	Change	F and *p* value
Time at the center in OFT at 4 weeks	Decrease in females	F = 10.68, *p* = 0.0024
Latency to grooming in SPT at 7 weeks	Increase in females	F = 4.655, *p* = 0.047
Distance traveled and average velocity in OFT at 7 weeks	Increase in females	F = 7.341, *p* = 0.0084 F = 7.030, *p* = 0.0098
Adipocyte area	Decrease in females	F = 6.105, *p* = 0.0244
TGF-β plasma levels	Increase in females	F = 11.45, *p* = 0.0015
CD4 + total T cells in blood	Increase in females	F = 4.443, *p* = 0.0405
CD4 + effector T cells in blood and spleen	Increase in females	F = 4.649, *p* = 0.0398 F = 6.710, *p* = 0.0127
IL-1β, Foxp3 RNA in the spleen	Increase in females	F = 15.08, *p* = 0.0004 F = 4.573, *p* = 0.0392
IL-17 RNA in spleen	Decrease in females	F = 4.603, *p* = 0.0389
TGF-β, C3, CD68, CD86, Arg RNA in brain	Increase in females	F = 11.31, *p* = 0.0025 F = 4,821, *p* = 0,0372 F = 11.11, *p* = 0.0032 F = 7.305, *p* = 0.0120 F = 4.337, *p* = 0.0473
CD206 RNA in brain	Decrease in females	F = 4.397, *p* = 0.0463
Astrocyte number	Increase in females	F = 6.771, *p* = 0.0163

Isolation stress- related differences		
Variable	Change	F and p value
Time in the light in LDBT at 4 weeks	Decrease in isolated	F = 11.23, *p* = 0.0038
Latency to grooming in SPT at 4 weeks	Increase in isolated	F = 9.951, *p* = 0.0035
Time immobile in FST at 7 weeks	Increase in isolated	F = 16.90, *p* = 0.0001
Grooming time in SPT at 7 weeks	Decrease in isolated	F = 12.30, *p* < 0.0001
Latency to grooming in SPT at 7 weeks	Increase in isolated	F = 8.784, *p* = 0.036
Time at the center in OFT at 7 weeks	Increase in isolated	F = 7.483, *p* = 0.0087
Time in the light in LDBT at 7 weeks	Decrease in isolated	F = 6.590, *p* = 0.0141
Adipocyte area	Decrease in isolated	F = 9.195, *p* = 0.0075
Food consumption	Increase in isolated	F = 6.486, *p* = 0.0142
Neutrophils in blood	Increase in isolated	F = 9.371, *p* = 0.0048
IFN-γ, IL-17, IL-10 RNA in spleen	Decrease in isolated	F = 4.709, *p* = 0.00367 F = 9.841, *p* = 0.0035 F = 7.833, *p* = 0.0083
IDO RNA in brain	Increase in isolated	F = 6.45, *p* = 0.00174
Microglia number	Increase in isolated	F = 11.38, *p* = 0.0022

Sex x isolation stress interaction		
Variable	Change	F and p value
Time of grooming at 4 weeks	Decrease in isolated males	*p* = 0.0263
Body weight	Decrease in isolated males	*p* < 0.0001
Corticosterone	Increase in isolated females	*p* < 0.0001
IL1-β RNA in brain	Increase in isolated females	*p* = 0.0113
Microglia total area	Increase in isolated females	*p* = 0.0463

## Discussion

Several models of chronic stress have been developed to reproduce depressive-like behaviors, which differ in the intensity and timing of the signs displayed, as well as in the degree of systemic and brain inflammation responses. This diversity is valuable when considering the heterogeneity observed in depressed and anxious patients, as it expands the available tools to better understand these psychiatric illnesses. Unfortunately, few studies have applied these paradigms to evaluate females, despite the well-established sexual dimorphism in immune function and brain connectivity.

Social isolation as a stress protocol has the advantages of being simple, less invasive, and, most importantly, capable of accurately replicating a human condition. It represents a chronic and profound form of psychological stress that significantly impacts mental health, with a lack of social support being a well-established risk factor for depression [39, 40]. Just a few years ago, the world experienced a global pandemic that forced us to isolate ourselves to prevent infection, and this context helped to highlight the importance of social interaction in human development and mental health.

In this work, we examined the effects of social isolation in both male and female mice, with a focus on depressive-like behavior, body weight, corticosterone levels, and biological alterations in the peripheral immune system and the brain.

Depressive- and anxiety-like behaviors in adult males have been previously reported after three [41], four [8, 42], and eight weeks of social isolation [43]. In females, however, the results have been scarce and more variable, depending on the time point analyzed. For example, no behavioral effects have been reported after four or five weeks of social isolation in mice or rats [17, 18], whereas another study has reported depressive-like behavior in the tail suspension test (TST) but not in the FST after eight weeks of social isolation [19]. Recent postmortem structural MRI studies have revealed marked sex differences in brain volume and tract density after two weeks of social isolation, with females showing greater changes in certain brain regions and fiber tracts [44], and alterations in fear memory extinction have been reported only in females. However, this study did not assess mood-related behaviors.

In our study, we detected depressive-like behavior after four weeks of isolation, through the splash test, observing increased grooming latency in males and females, and decreased grooming time only in males. After seven weeks, depressive-like behavior was evident in both sexes through the FST and splash test, suggesting that females may develop depressive-like symptoms at later stages than males do, highlighting the importance of the chronicity of the stress protocol. Anxiety-like behavior was detected in both sexes after four and seven weeks of isolation by LDBT, but not by OFT. Intriguingly, both males and females, after seven weeks of isolation, increased their time at the center of the arena, compared to the controls, which is the opposite of what it could be expected. As OFT analyzes the struggle between the instinct to explore and the fear of open illuminated spaces, our results could suggest that chronically isolated mice have higher exploratory motivation, as a coping mechanism to isolation.

Interestingly, we observed that isolated males lost weight during the protocol, which is consistent with other stress models [27], whereas females did not. In fact, although not significantly different, a clear trend of increased body weight in females can be detected. This differential body weight pattern was also reported by Challa et al. [45] and this is particularly interesting because female mice are known to be resistant to weight gain in the context of diet-induced obesity, for instance [46, 47]. Because food consumption increased in both sexes, this cannot explain the body weight difference. One factor that consistently has been shown to promote weight gain by increasing food consumption, altering adipose tissue distribution and accumulation, and decreasing metabolic rate is glucocorticoids (cortisol in humans and corticosterone in mice) [48]. In our study, isolated females presented higher corticosterone levels than controls did, with no differences in males, similar to findings in male rats [49] or in male mice after twelve weeks of isolation [10]. Our results indicate increased HPA axis activity in socially isolated females. In contrast, Smolensky et al. reported weight reduction in both sexes without changes in corticosterone levels after four weeks of isolation [17]. Altogether, the results suggest a possible link between body weight changes under stressful conditions and corticosterone levels, which could explain the different effects on body weight observed between females and males after eight weeks.

Consistent with previous reports [17], female mice were shown to have higher basal corticosterone levels than males, potentially due to the enhancing effect of estradiol on HPA axis responses [50]. Although the estrous cycle and pre-stress baseline measurements were not monitored in this study, an acknowledged limitation, previous research in rats indicates that depressive-like behaviors induced by chronic social isolation are not dependent on estrous stages [51]. Pre-stress baseline measurements, however, would have provided a clearer picture of HPA axis dynamics.

Although stress is widely recognized to disrupt homeostatic control of the immune system and systematic reviews and meta-analyses link depression to an inflammatory state [52], we did not observe differences in plasma IL-6 levels, unlike other studies that used different psychological stress paradigms [53]. Moreover, there were no changes in circulating CD4^+^ T cells, CD4^+^ effector T cells, or Tregs cells due to isolation, despite previous reports of reduced Tregs cells in males subjected to chronic restriction stress [27, 32] or in MDD patients [54–56], suggesting that these cell populations may change depending on the type of stress. However, sex-related differences were observed for some of these populations, finding increased frequencies of CD4 + T cells with an effector phenotype in the blood and spleen of female mice. These results are consistent with previous literature showing females have a basal increased frequency of several innate and adaptive immune cells, being able to activate immune responses faster than males [13], which translates into a protective response against infections but also poses a risk to autoimmunity. This differential immune profile in females can be explained by the activator effect of estrogen, and the expression of some immune-related genes present on X chromosome [57].

In our study, we detected increased neutrophil frequency in isolated mice, which is consistent with findings in depressed patients and in male mice subjected to repeated social defeat [58]. Chronic unpredictable stress, which is another common stress model, has also been reported to increase leukocytes, monocytes, and neutrophils in females [59].

In the spleen, we found no differences due to stress in total CD4^+^ T cells, Treg cells, Th1, and Th17 cells. Some studies have reported increased total CD4^+^ and CD8^+^ T cells in male mice under chronic unpredictable stress [60], whereas no effects on CD4^+^ T cell frequency have been detected in male mice subjected to chronic restraint [61] or social defeat [62], suggesting again that changes in these populations are sensitive to the type of stress. However, we observed decreased expression of IFN-γ, IL-10, and IL-17 mRNA in stressed mice, which could indicate a dysregulation of adaptive immune responses. Although there is no clear consensus regarding the effect of stress on IL-10 [63], increased IL-10 has been linked to reduced depressive-like behavior, so lower levels of IL-10 could be maintaining depressive-like behavior [64–66].

Expression analysis of inflammatory mediators in the hippocampus showed increased expression of IDO in isolated mice of both sexes. IDO upregulation has been documented in rat and mouse models of chronic unpredictable stress, leading to increased levels of the excitotoxic molecule quinolinic acid [67, 68]. CD86 expression is known to increase in proinflammatory reactive microglia [69]; however, although a trend is observed for females, no statistical differences were detected after social isolation stress. IL-1β, on the other hand, a major proinflammatory cytokine, was found to be increased only in isolated females. Morphological analysis confirmed increased Iba1^+^ microglial numbers in the dentate gyrus of the hippocampus in both sexes, while an increase in the total area occupied by microglia was detected in isolated females. These morphological changes, together with increased expression of IL-1β suggest higher microglial reactivity in females in response to social isolation stress.

A previous study on two-week social isolation revealed an increased branch territory in the dorsomedial hypothalamus and increased branch territory, cell volume, and branch endpoints in the hippocampus of males but not females [25]. Interestingly, these results are consistent with the reported early appearance of depressive-like behavior in males.

Regarding astrocyte numbers, we did not observe changes after social isolation exposure, although multiple studies have shown that other chronic stress paradigms markedly decrease the expression of GFAP [70–74] and reduce the number of GFAP-immunoreactive cells [75]. However, some studies report no change [71] or even increased GFAP levels in the hippocampus [76, 77]. These discrepancies may reflect differences in the type or intensity of the stressor or the timing between stress exposure and astroglia assessment [76, 77]. Interestingly, sex differences were observed for astrocytes number, finding a higher number in the dentate gyrus of females, as previously reported [78]. The increase in the presence of astrocytes, which is also influenced by estrogen, could suggest a greater capability of the female brain to adapt to environmental changes and protect neurons under stressful conditions; however, it might also pose a higher risk for neuroinflammation, as differences in their capacity to produce proinflammatory cytokines have also been reported [79, 80].

The highest number of neuroinflammation signs observed in isolated females coincided with elevated corticosterone levels, so it may be tightly linked to heightened HPA axis activation and glucocorticoid signaling. Microglia express glucocorticoid receptors and can be directly modulated by circulating corticosterone, leading to phenotypic shifts toward a proinflammatory state under chronic stress conditions [81]. Notably, estrogen can potentiate HPA axis responsiveness [50]. The fact that males did not show changes in corticosterone might reflect a different phase of HPA axis dysfunction beyond the hypercorticosteronemia state, suggesting an attempt to restore homeostasis. These sex-dependent neuroimmune-endocrine interactions underscore the need to account for hormonal status and glucocorticoid sensitivity when interpreting neuroinflammatory outcomes in stress models.

In summary, distinct biological responses were observed between the sexes, highlighting sex-specific responses to social isolation stress. Such differences should be considered when refining biological diagnostics and designing pharmacological interventions for mental illness.

It would be relevant to explore the reversibility of the observed phenotype in both sexes, assessing whether behavioral and immunological changes persist or can be resolved after resocialization, for instance.

## Conclusions

After seven weeks of social isolation, although both sexes exhibit depressive-like behavior and neuroinflammatory signs, such as elevated neutrophils in circulation, decreased IL-10 expression in the spleen, higher expression of IDO in the hippocampus, and higher microglia number, sexual dimorphism in response to this type of stress is detected. Isolated males lower their body weight and do not show altered corticosterone levels, while isolated females increase corticosterone levels, IL1-β expression in the hippocampus, and microglia total area. Overall, our findings highlight the complexity and sex-specific nature of behavioral, hormonal, and immune responses to chronic social isolation. Future studies should aim to deepen the mechanistic understanding of these sex differences, particularly focusing on the role of glucocorticoid signaling, neuroimmune interactions, and regional brain vulnerability.

Integrating omics approaches and longitudinal analyses will be key to disentangling the temporal and molecular cascades leading from chronic stress to depressive-like states, contributing to the development of more precise and effective interventions for stress-related disorders.

## Supplementary Information

Supplementary Material 1

## Data Availability

The datasets used and/or analyzed during the current study are available from the corresponding author on reasonable request.
