# Supplementary material for: Prolonged social isolation promotes depressive-like behavior in male and female mice, with sex-related differences in the stress response
Source: Biol Sex Differ. 2026 Mar 12;17:94. doi: 10.1186/s13293-026-00874-0 (PMC13130425; doi:10.1186/s13293-026-00874-0)
Supplement: Supplementary file 1 — Supplementary Material 1 [file 13293_2026_874_MOESM1_ESM.docx]

Supplementary Table 1

| **Gene** | **Forward** | | **Reverse** |
| --- | --- | --- | --- |
| **TNF-ɑ** | | 5’-CTCAGCCTCTTCTCATTCCT-3’ | 5’-CTTGGTGGTTTGCTACGAC-3’ |
| **IL-6** | | 5’-GTTGCCTTCTTGGGACTGAT-3’ | 5’-GCCATTGCACAACTCTTTTC-3’ |
| **IL-1β** | | 5’-GATGAGAGCATCCAGCTT-3’ | 5’-GTCACAGAGGATGGGCTCTT-3’ |
| **TGF-β** | | 5’-TTTTGCTCCTGCATCTGGT-3’ | 5’-CCTGGTACTGTTGTAGATGGAA-3’ |
| **IL-10** | | 5’-GACAATAACTGCACCCACTTC-3’ | 5’-ACCCAAGTAACCCTTAAAGTCC-3’ |
| **Foxp3** | | 5’-CTGCTGGCAAATGGAGTCTG-3’ | 5’-CCAGAGACTGCACCACTTCT-3’ |
| **IFN-γ** | | 5’-GCCAAGTTTGAGGTCAACAACC-3’ | 5’-ATCTCTTCCCCACCCCGAAT-3’ |
| **IDO** | | 5’-AGAGCTCGCAGTAGGGAACA-3’ | 5’-GGCTAGAAATCTGCCTGTGC-3’ |
| **C3** | | 5’-AACTACCGTGAACAGGAGGA-3’ | 5’-TGGAAGTAGCGATTCTTGGC-3’. |
| **CD68** | | 5’-CGCTTATAGCCCAAGGAACA-3’ | 5’-CCACGTGTAGTTCCCAAGAG-3’ |
| **CD86** | | 5’-ACTCCTGTAGACGTGTTCCA-3’ | 5’-GCCCAAATAGTGCTCGTACA-3’ |
| **CD206** | | 5’-TGTACTTTGAGTGGAGTGATGG-3’ | 5’-GGGTCACCTTTCAGCTCAC-3’ |
| **Arg-1** | | 5’-TGCTCACACTGACATCAACA-3’ | 5’-TACACGATGTCTTTGGCAGA-3’ |
| **IL-4** | | 5’-CCAGCTAGTTGTCATCCTGC-3’ | 5’-CCTCGTTCAAAATGCCGATG-3’ |
| **18S** | | 5’-GCCCGAAGCGTTTACTTTGA-3’ | 5’-TTGCGCCGGTCCAAGAATTT-3’. |

List of primers used in qRT-PCR.
